# High-amplitude electrical stimulation can reduce elicited neuronal activity in visual prosthesis

**DOI:** 10.1038/srep42682

**Published:** 2017-02-17

**Authors:** Alejandro Barriga-Rivera, Tianruo Guo, Chih-Yu Yang, Amr Al Abed, Socrates Dokos, Nigel H. Lovell, John W. Morley, Gregg J. Suaning

**Affiliations:** 1 Graduate School of Biomedical Engineering, UNSW, Sydney, 2052, Australia; 2 School of Medicine, Western Sydney University, Sydney, 2753, Australia; 3 School of Medical Science, UNSW, Sydney, 2052, Australia; 4 Sydney Medical School, University of Sydney, 2000, Australia

## Abstract

Retinal electrostimulation is promising a successful therapy to restore functional vision. However, a narrow stimulating current range exists between retinal neuron excitation and inhibition which may lead to misperformance of visual prostheses. As the conveyance of representation of complex visual scenes may require neighbouring electrodes to be activated simultaneously, electric field summation may contribute to reach this inhibitory threshold. This study used three approaches to assess the implications of relatively high stimulating conditions in visual prostheses: (1) *in vivo*, using a suprachoroidal prosthesis implanted in a feline model, (2) *in vitro* through electrostimulation of murine retinal preparations, and (3) *in silico* by computing the response of a population of retinal ganglion cells. Inhibitory stimulating conditions led to diminished cortical activity in the cat. Stimulus-response relationships showed non-monotonic profiles to increasing stimulating current. This was observed *in vitro* and *in silico* as the combined response of groups of neurons (close to the stimulating electrode) being inhibited at certain stimulating amplitudes, whilst other groups (far from the stimulating electrode) being recruited. These findings may explain the halo-like phosphene shapes reported in clinical trials and suggest that simultaneous stimulation in retinal prostheses is limited by the inhibitory threshold of the retinal ganglion cells.

Retinal dystrophies such as retinitis pigmentosa induce progressive loss of photoreceptors, resulting in profound vision impairment. Visual neuroprostheses aim to restore patterned vision to those with vision loss by electrically stimulating the surviving neurons in the visual system. Different stimulation sites along the visual pathway have been investigated for their applicability to visual implants including, the retina^1^,^2^,^3^, the optic nerve^4^,^5^, the lateral geniculate nucleus (LGN)^6^, and the primary visual cortex^7^. Although thalamic and cortical stimulation could treat not only those conditions affecting photoreceptors, but also other diseases such as glaucoma or diabetic retinopathy, direct retinal stimulation has several advantages over these approaches, as the retina is the natural interface of the visual system. These include retinotopic mapping of stimuli^8^ as well as providing simplified surgical access, particularly in the suprachoroidal space^9^.

Electrical stimulation of surviving retinal ganglion cells (RGCs) has proved that restored visual perception is feasible, demonstrating palpable outcomes in human subjects^8^. Over the last 15 years, significant progress has been made by several groups throughout the world in the design, development and testing of retinal prostheses. Given the encouraging initial results in human trials, it is highly probable that retinal prostheses will be the first widely-adopted therapeutic option for restoring sight to those blinded by neurodegenerative diseases for which there are no effective therapies currently. Clinical investigations are showing promising results with recipients being able to perceive visual patterns and perform mobility tasks in daily life^10^. At present, visual prosthetic devices provide a visual acuity approaching that required to restore functional sight^11^,^12^,^13^, with current technologies able to place up to 1500 electrodes over a 3 × 3 mm^2^ area. However, the resolution achieved with these devices can be compromised when delivering concomitant stimulation at multiple sites as a result of electric and neural cross-talk^14^,^15^. This can be explained in terms of a wide electric field spread from single electrodes, causing excessive stimulation levels by summation of overlapping electric fields^16^. Although concomitant stimulation of contiguous electrodes has important limitations, it may still be required to present complex visual scenes to the recipients of visual prostheses^17^,^18^. The undesired effects relating to cross-talk will be amplified in prostheses with densely packed electrode arrays where placement is relatively far from the neural targets as in the case of supra-choroidal stimulation.

It is known that electrical stimulation can produce both activation and inhibition of the RGCs. Boinagrov and co-workers explained the inhibitory effect as sodium current reversal under strong stimulation conditions and demonstrated the existence of a so-called upper threshold *in vitro,* beyond which no spike can be elicited^19^. This threshold appears below harmful stimulation levels and not too far above the activation threshold. However Rattay proposed, in accordance with the classical approach^20^, anodal block as the reason for this neural inhibition^21^. Traditionally, single-pulse stimulus-response relationships have been considered as purely monotonic functions, i.e. non-decreasing spike numbers with respect to increasing current amplitude, and have been fitted to sigmoidal relationships^15^,^22^,^23^,^24^. Based on previous experimental studies on electrical interference in visual prostheses^15^,^25^, the authors hypothesize that additive effects of overlapping electric fields can inhibit the ability to evoke action potentials (APs) in the RGC regions close to stimulating electrodes^26^. As a result of this inhibition, non-monotonic spike numbers are expected in response to increasing levels of stimulation current.

Neural inhibition in response to high levels of retinal stimulation has been demonstrated in this study from three different points of view: (1) *in vivo*, (2) *in vitro* and (3) *in silico*. Firstly, in anesthetized cats, seven hexagonally-arranged electrodes in a suprachoroidal electrode array were activated simultaneously while penetrating electrodes recorded the evoked responses from the primary visual cortex. Furthermore, murine retinal preparations were electrically stimulated through a single electrode using monophasic pulses at different locations, with evoked APs recorded using the whole-cell patch-clamp technique. Finally, a population-based RGC computational model was developed to examine the response of a RGC layer and to explain the experimental outcomes.

## Results

### *In vivo* electrophysiology

Seven circular electrodes from a suprachoroidal visual prosthesis, arranged in a hexagonal pattern, were activated simultaneously using current amplitudes of between 0 and 600 μA. A minimum of 43/96 and a maximum of 92/96 cortical channels were considered to be responding as defined in the materials and method section. Responses were then automatically classified as monotonic, saturated, or non-monotonic. Other channels did not show spiking rates above the spontaneous spiking rate and therefore were considered as non-responding channels. Overall, 13.8% of those channels that were found to be responding could not be classified as monotonic, saturated or non-monotonic. These results are summarized in Table 1.

**Figure 1:**
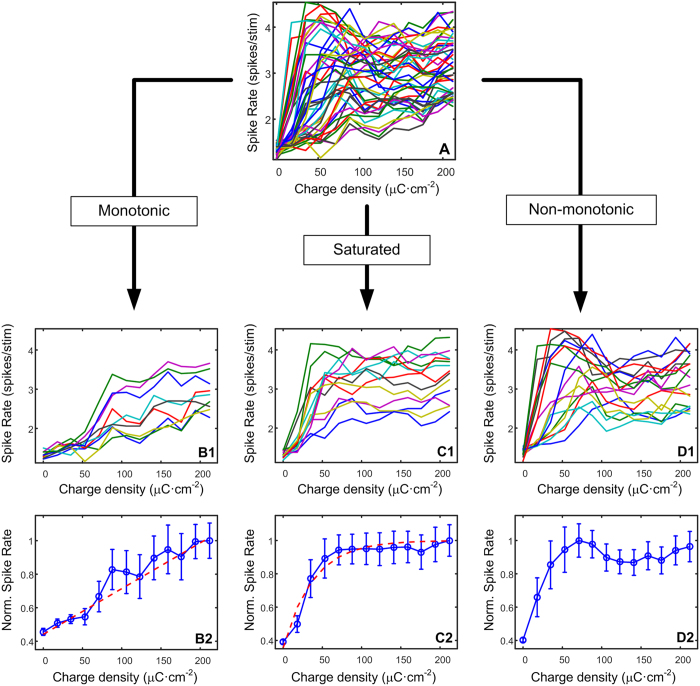
Example of overall responses obtained from one cat. Panel A shows the spiking rate of all responding channels. B1, C1 and D1 show the spiking rate of monotonic, saturated and non-monotonic channels respectively. B2, C2 and D2 illustrate the normalized average of monotonic, saturated and non-monotonic channels respectively using a confidence level of 95% for the error bars. Note that the dashed line in B2 and C2 represent the linear and increasing exponential decay fit respectively.

The P_50_ activation thresholds, defined as the mid-level point of the sigmoidal curve fits, were calculated, by (1) taking into account the average response of all responding channels, and (2) by considering only the average of those channels classified as saturated. The P_50_ values obtained were 36.4 ± 19.9 and 35.7 ± 13.4 μC·cm^−2^ respectively (n = 4). The ANOVA analysis revealed that both threshold measures were similar (p-value = 0.955). These current densities did not show statistically significant differences when compared to single electrode stimulation (p-value = 0.505), as in a previous report where the sample size was also four^15^. Figure 1 shows an example of spike rates in responding channels and the different profiles obtained after profile classification.

**Figure 2:**
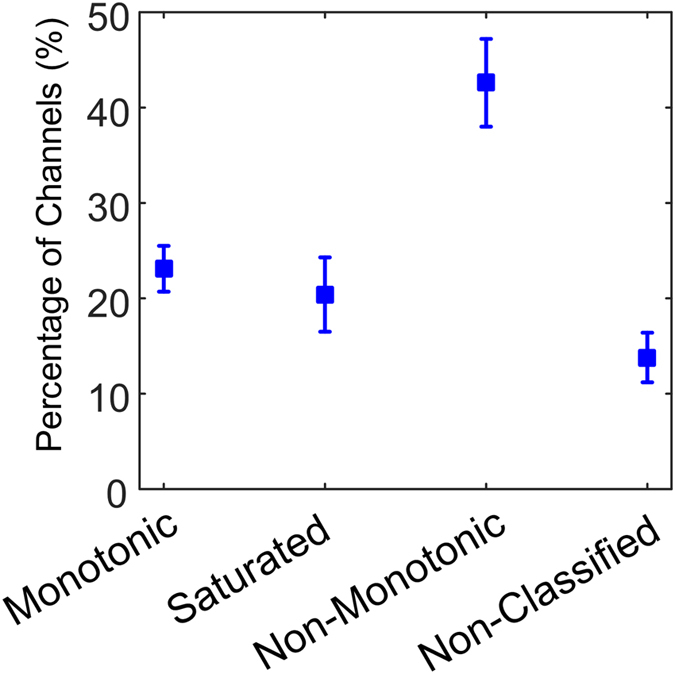
Overall (n = 4) percentage of response type relative to the total number of responding channels per cat. The number of non-monotonic channels was significantly greater than any other type (p-value < 0.05). Bars represent the standard error of the mean.

The overall proportion of channels classified as non-monotonic was significantly greater than monotonic (p-value = 0.007) or saturated (p-value = 0.009), and overall was as more than both of these together (Fig. 2).

**Figure 3:**
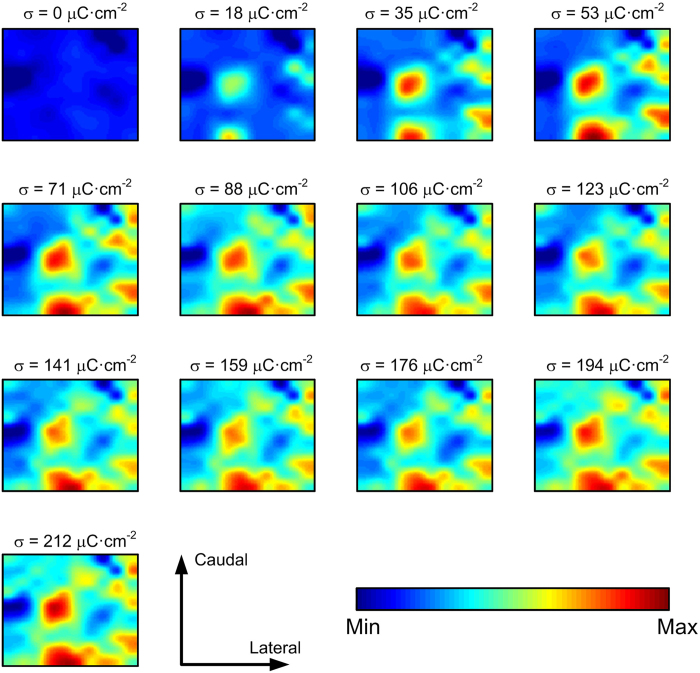
A series of cortical activation maps from the same animal is shown for different stimulating current levels. The spike rate was normalized between the base line level (blue) and the maximum activity (dark red). Images were smoothed using a 3 × 3 Gaussian filter with σ = 0.2. Current densities (σ) ranged between 0 μC⋅cm^2^ to 212 μC⋅cm^2^ when seven electrodes, arranged in a hexagonal pattern, were activated simultaneously. The sequence illustrates regions which exhibit a non-monotonic profile such as the area around the centre of each map.

Normalised cortical activation maps for each stimulation level are shown in Fig. 3. These cortical maps illustrate the overall spiking rate recorded in the primary visual cortex for all 96 channels. Some regions remained inactive regardless of the intensity of the electrical stimulus. These correspond to non-responding channels. In other regions, such as those near the centre of the array, a non-monotonic trend is evident.

### *In vitro* electrophysiology

**Figure 4:**
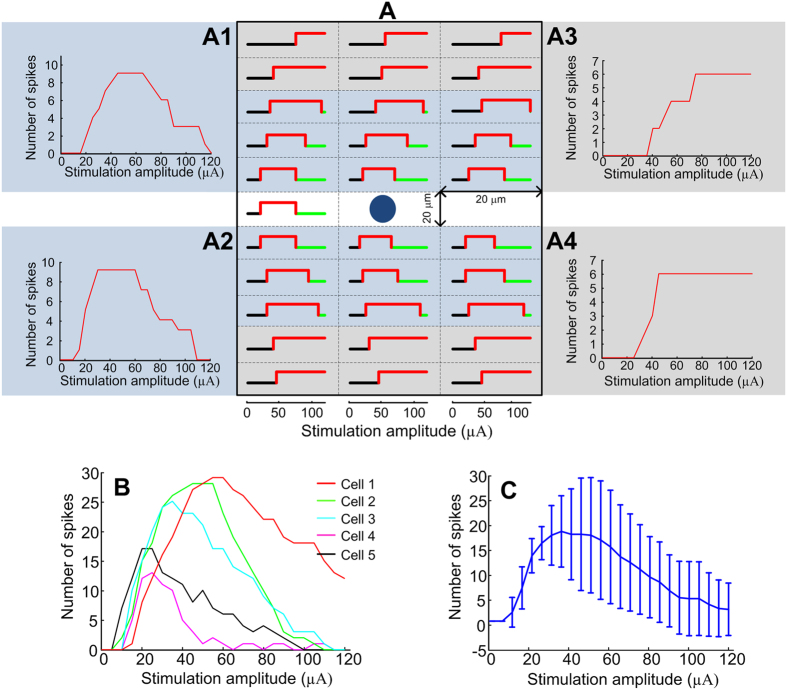
Overall responses obtained after placing the stimulating electrode at different locations separated laterally by 20 μm. Panel A illustrates the spike elicitation (red) and inhibition (green) curves as a function of stimulation amplitude plotted over tested locations in one cell. The centre blue dot indicates the soma of the patched RGC. Panels A1 and A2 illustrate non-monotonic spike counts when the stimulating electrode was placed near the soma. Panels A3 and A4 show saturated responses when the electrode was placed furthest from the soma. Panel B illustrates the spike count including all 31 locations, as shown in A, for each cell under study. Panel C shows the overall spike count as a function of the stimulating amplitude (n = 5). Error bars indicate standard deviation.

In order to understand how a population of the same RGC type responds to supra-threshold stimulation, a small stimulating electrode (SE) was positioned epiretinally and moved to several locations, each with a lateral separation of 20 μm from the previous site. The results obtained can be considered equivalent to the case of a fixed, stationary SE and several RGCs of the same type distributed around. For each stimulation amplitude, the patched RGC showed either no response (due to a sub-threshold or an excessive stimulation level) or elicited a spike (produced by adequate supra-threshold stimulation). Figure 4A illustrates the occurrence of an AP as a response to increasing stimulation amplitudes with the SE positioned at 31 different locations. It can be seen that the stimulation amplitude at which the spikes were elicited or inhibited depends on the spatial location of the SE, that is, the distance between the electrode and the cell.

To demonstrate multi-unit profiles, spikes elicited at different regions were grouped together. The resulting profiles were then classified as saturated or non-monotonic using the same criteria defined during the *in vivo* experimentation. Two main regions were considered: one with SE close to the patched RGC (or RGCs close to the stimulating electrode) and the other with SE farther away from the patched RGC (or RGCs distal to the stimulating electrode). In the former case, non-monotonic response curves were observed as shown in Fig. 4A1,A2. In the latter case, the response curves reached saturation as shown in Fig. 4A3,A4. Likewise the overall profile from all SE positions tested resulted in a non-monotonic response, comparable to those reported *in vivo* (see Fig. 1).

To demonstrate the general occurrence of non-monotonic responses in a population of RGCs, the total spike count over all SE positions for each cell studied is shown in Fig. 4B. Each of these curves was classified as non-monotonic, as well as the overall response (n = 5) illustrated in Fig. 4C.

### Electric potential

**Figure 5:**
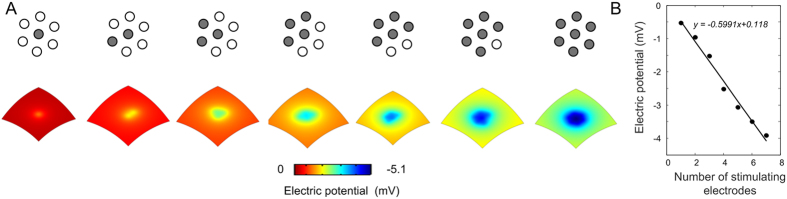
Panel A shows electric potential, obtained using the finite element method following simultaneous activation of several electrodes as shown in the top row. Gray and white circles represent active and non-active electrodes respectively. Panel B illustrates the value of the electric potential underneath the centre electrode of the hexagon as a function of the number of electrodes simultaneously activated with a cathodic pulse.

The electric potential was computed over a 2D surface (~6 mm × ~6 mm) representing the location of target neurons, 400 μm from the stimulating hexagon using the finite element method (FEM) as described in the methodology. The electric potential profiles due to increased number of active stimulating electrodes are illustrated in Fig. 5. Computational simulations indicate a linear summation (r^2^ = 0.99) of the potential directly underneath the central electrode as well as in the proximity of the hexagon (r^2^ ≥ 0.97). The slope of the linear regression beneath the central electrode, as in Fig. 5B, was approximately −0.6 mV/Electrode, representing an extra drop of approximately 0.6 mV in the electric potential per extra concurrently activated electrode for a stimulating current of 200 μA. The decay in the electric potential would be much larger as the stimulating current increases.

### Population-based computational analysis

**Figure 6:**
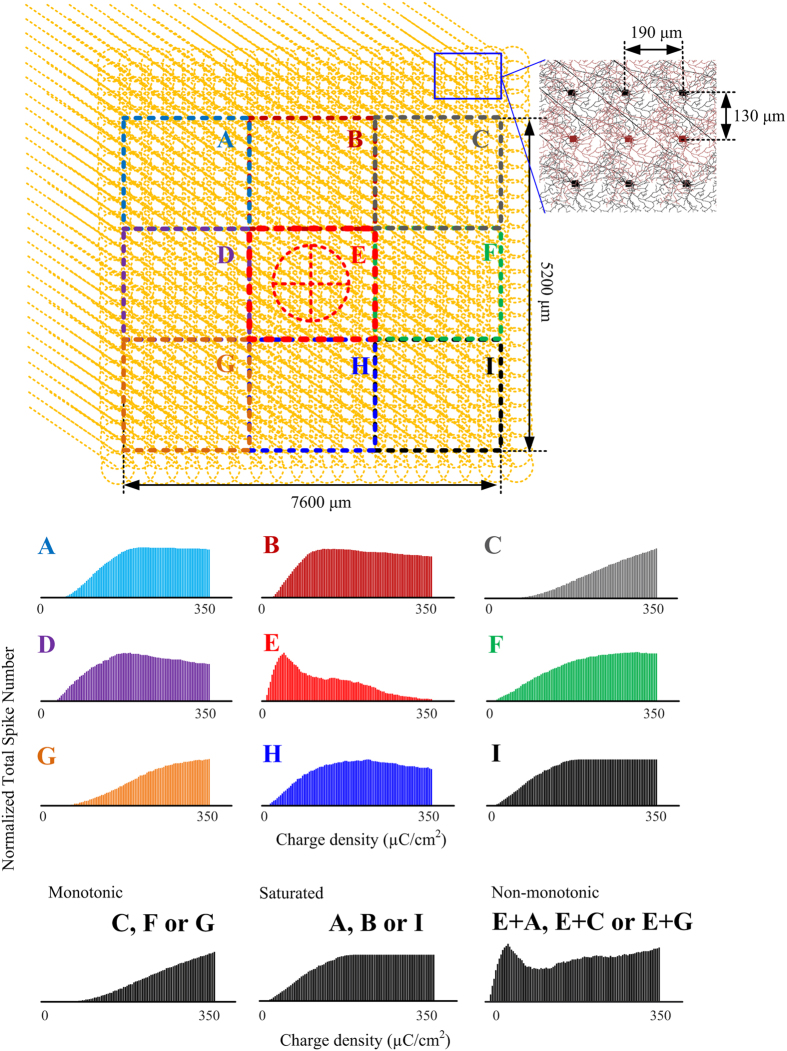
Upper panel: Population-based RGC model. Nine functional regions (**A–I**) were classified around the stimulation electrode (red circle). Each region includes 13 × 13 cells. In total 39 × 39 cells were simulated. RGCs were uniformly distributed on a rectangular grid with 190 μm lateral and 130 μm longitudinal distances between neighbouring cells. Lower panel: Stimulus-response relationship reproduced by population-based RGC model. Total spike number of nine RGC populations was obtained in each functional region shown in the upper panel. All stimulation conditions were identical to those conducted *in vivo*. The charge density ranged from 0 to 350 μC/cm^2^. Three classified response patterns (monotonic, saturated and non-monotonic) were able to be reproduced by particular RGC populations or their combination, as indicated in the lower panel.

A population of approximately 1500 RGCs was mathematically modelled and implemented computationally. A single electrode equivalent in area to the hexagonal unit used *in vivo* (7 × 0.113 mm^2^) was activated using charge-balanced, symmetric, biphasic constant-current pulses with phase duration 400 μs and inter-phase delay 10 μs in the same fashion as the *in vivo* experimentation. Responses to different stimulating current densities were obtained, with retinal activation maps shown in Fig. 6. Initially, the area directly underneath the electrode was activated. However, as the stimulation current increased, more peripheral neurons started firing whereas more central cells were gradually inhibited.

The population-based simulations revealed that cell clusters near the stimulation electrode (region E) exhibited non-monotonic stimulus-response profiles. Likewise cell populations surrounding the electrode showed either monotonic (regions C, F, G and I) or saturated (regions B, D and H) response profiles. Interestingly, the combination of certain populations (e.g. E + C, E + D) was able to predict experimentally-recorded non-monotonic stimulus-response relationships *in vivo*.

## Discussion

Clinical trials have shown that parallel and interleaved stimulation may be complementary paradigms in retinal neurostimulation^27^. Large and bright objects presented in a visual scene may require several neighbouring electrodes to be activated simultaneously. Although the interaction between adjacent electrodes can be used, for example, to reduce activation thresholds in the vicinity of an active electrode^15^ or to evoke intermediate percepts^25^, the beneficial effects of field overlapping can be achieved with sub-threshold stimulus levels. Nonetheless if a group of electrodes are activated simultaneously using supra-threshold current densities, deleterious effects can result such as a reduced visual acuity^16^ or an undesired neural inhibition^26^ as shown in this study. The possible mechanisms underlying the inhibitory effect of a large electric field were studied both *in vitro* and *in silico*^19^,^21^,^28^. Further study of the RGC distal axons will ultimately be required in order to reveal the detailed mechanisms at play here.

Results presented here agree with a recent publication by Abramian and co-workers^26^, who demonstrated non-monotonic responses in a grid of RGCs using a computational model based on the Fohlmeister formulation of ionic currents^29^. To the authors’ knowledge, this current study is the first to show non-monotonic responses to *in vivo* retinal electrostimulation, whereby firing rates reached a maximum value and then clearly declined with increased stimulus intensities in sighted cats. Retinal degeneration models^30^ or the use of synaptic blockers^31^ should be contemplated in future research. Note that according to Elfar *et al*.^32^ cortical responses recorded within the first 20 ms following retinal stimulation may arise from axonal and somatic RGC responses whereas delayed responses may occur due to indirect network-driven RGC activation subsequent to the stimulation of bipolar cells and/or photoreceptors^33^. This hypothesis is commonly established among scientists working in visual prosthetics^15^,^24^,^25^,^34^. However, in this study, a more restrictive time window was chosen to ensure that only direct activation was accounted for exclusively^35^.

Higher current densities, up to 300 μC·cm^−2^, have been previously used in similar experiments^24^,^25^. Note that the net injected charge in these trials was considerably lower: in this study, seven electrodes were simultaneously activated within a small retinal region resulting in larger net charge injection. According to Matteucci *et al*.^15^, a secondary concurrent sub-threshold monopolar stimulus, delivered 2 mm away from a primary stimulation site, can lower the activation threshold by approximately 20% of the interfering current. Here, the concomitant activation of seven densely arranged electrodes, spaced apart by less than 1 mm, enhances the electric field where the individual fields created by each electrode overlap, as illustrated in Fig. 5. This produces significant electric and neural cross-talk effects which are difficult to separate from each other. These effects would be enhanced when the distance between electrodes and the neural targets decreases, as in the case of epiretinal and subretinal neuroprosthesis approaches. Perhaps non-monotonic responses were not previously reported *in vivo* because the field created by a single electrode activates a smaller retinal region. In this study, the proportion of non-monotonic responses was similar to that of the combined monotonic and saturated responses, which are all non-decreasing curves. In case of single electrode stimulation, a lower number of non-monotonic cortical responses can be expected, as well as this number to be mediated by the stimulus strength – with increasing non-monotonic responses corresponding to increasing stimulus strength.

As previously stated, *in vitro* results obtained by moving the stimulating electrode around a patched RGC can be considered equivalent to probing a local population of RGCs of the same type. This has been shown using a computational model of a large population of RGCs. In both cases, non-monotonic curves indicated that inhibition would occur for large stimulation amplitudes which in turn, would be reflected in the primary visual cortex. *In vitro* and *in silico* experiments showed that groups of cells far from the SE exhibit non-decreasing profiles. The reason that most patched RGCs showed mainly non-monotonic curves was possibly due to the spatially restricted SE locations tested (maximally 100 μm between the patched cell and the SE). Although in this study pure monotonic curves were not evidenced *in vitro*, computational analysis suggests that such monotonic responses are feasible by extending the probed area beyond the limits defined in this work.

Based on previous computational models^16^, and the estimation of the electric potential in this study, a large single electrode with greater stimulating current levels represents a simplified approach to the electrode scenarios utilized in this study during the population-based analysis.

The computational analysis and the *in vitro* results presented in this paper suggest that inhibition of RGCs in different retinal areas can explain the cortical responses obtained *in vivo*. However, the amount of information processed at the lateral geniculate nucleus (LGN) is still far from being completely identified^7^,^36^,^37^ and may also play a role. Thus, the absence of bell-shaped curves *in vivo*, as in Fig. 6E, may be explained as a combination of relatively large retinal regions contributing to multiunit responses in area 17 of the visual cortex, to a certain degree of visual processing performed at the sub-cortical structures, or both.

Several possible limitations exist in the present population-based computational model. First, RGCs were uniformly distributed with a cell density of approximately 40 cells/mm^2^ and their axons were running parallel to each other. Furthermore, the model assumed independent firing of neighbouring RGCs. Secondly, the reported impact of gap junction coupling on RGC behaviour^38^ should be incorporated into a detailed network model. Finally, due to limited experimental data on anisotropies of the retinal layers, the present model assumes homogeneous retinal tissue. As mentioned before, other authors have considered population-based computational models of RGCs to predict retinal responses to electrical stimulation^26^,^39^. However, the model presented here extends the results to a broader retinal area and explains the responses to strong electrical stimulation as observed *in vivo*.

The results presented in this study support previous research which highlighted a reduction in visual acuity as a consequence of parallel stimulation^16^. This work also showed that excessive electrical stimulation may reduce cortical activation, especially that resulting from electric crosstalk in tightly arranged electrode arrays. This inhibitory effect would be more relevant in other retinal approaches, whereby the stimulating electrodes are placed near the RGCs; a closer proximity to the neural targets allows more focused and contained electric fields which could partially relieve this effect. The results presented here can explain the halo-like phosphene shape reported in clinical trials in terms of local inhibition occurring in close proximity to the electrode being activated^40^. According to a recent *in silico* study published by Werginz and Rattay^41^, strong stimulation with monophasic anodic pulses can result in reversal of calcium flow in retinal bipolar cells. This may explain some of the widely variable phosphenes elicited by retinal electrostimulation. Further research in electric field manipulation must address these limitations, particularly in the way that the electric field can be contained and focused and through selective activation using, for example, high frequency stimulation^42^. *In vivo* electrophysiology studies with models of retinal degeneration and the use of synaptic blockers may help in understanding the role of activation of the retinal network in similar scenarios. In particular, single unit recordings from the superior colliculus or the LGN may shed further light, since the neural information in these visual centres originates directly from the RGCs.

## Materials and Methods

### Ethics Statement

Approval was obtained from the Animal Care and Ethics Committee of the University of New South Wales (UNSW), Australia. All procedures were carried out in compliance with the Australian Code of Practice for the Care and Use of Animals for Scientific Purposes and with the ARVO statement for the use of animals in ophthalmic and vision research.

### *In vivo* electrophysiology

#### Stimulating electrode array

**Figure 7:**
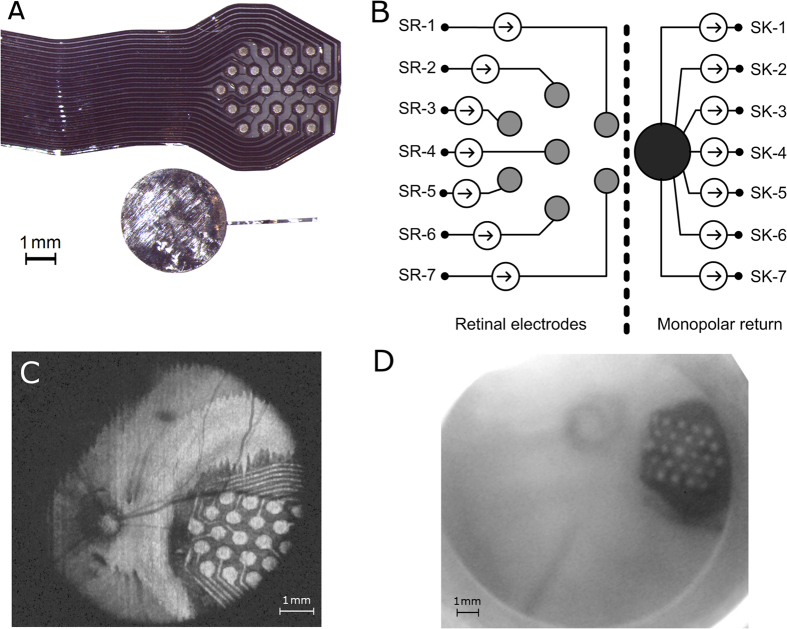
Panel A illustrates the 24-channel retinal electrode array and the return electrode used during *in vivo* electrophysiology. Each electrode within the array has a diameter of 380 μm (area 0.113 mm^2^) and centre-centre spacing of 730 μm. The return electrode was approximately 3.5 mm in diameter. Panel B illustrates connections between the neural stimulator and the electrode array. Seven electrodes arranged hexagonally were connected to the source (SR-i) of seven independent current sources. The monopolar return electrode was connected to the sink of all channels (SK-i). Panel C is an optical coherence tomography image obtained after implantation. The voxels containing information on the electrode array were projected into a 2-dimensional image. Panel D shows an infrared image of the fundus of the same implanted eye.

A 24-channel electrode array was fabricated at the Graduate School of Biomedical Engineering, UNSW, Australia, as described previously^43^. Briefly, a 25 μm thick platinum foil was laminated to a polydimethylsiloxane (PDMS) substrate and mechanically strengthened by a polyethylenetherephalate (PET) mesh. The electrode profiles were then cut using a laser micromachining technique followed by the application of a covering layer of PDMS. Afterwards, the electrodes were exposed using a 193 nm wavelength excimer laser and the surface was roughened through structured laser interference patterning to increase the effective area of the electrode, as described by Green *et al*.^44^. Each circular electrode had a diameter of 380 μm (area 0.113 mm^2^) and a centre-to-centre distance of 730 μm. The electrodes were arranged hexagonally as illustrated in Fig. 7. The stimulating electrode array was interfaced to a custom 14-channel stimulator^45^, using a switch matrix (NI PXI-2532, National Instruments Corporation, Austin, Texas, USA) to facilitate further electrode configurations. The switch matrix allows connecting any individual electrode to one or several stimulating channels without rewiring^46^. Each electrode was therefore driven, relative to a distant monopolar electrode, by an independent push-pull current source with mismatch less than 10% of the total current, as shown in Fig. 7B. The accumulation of charge as a result of any current mismatch was subsequently dissipated between stimulation pulses by means of inter-stimulus shorting of all electrodes to a common potential. The impedances of the electrode-tissue interfaces were monitored by measuring the voltage waveforms using a digital multi-meter (PXI-2532, National Instruments Corporation, Austin, Texas, USA).

#### Recording electrodes

In the first stage, a 2.3 mm diameter platinum ball electrode was used to record local field potentials from the surface of the striate cortex. This served to localize the approximate area of cortical representation of the retinal space being stimulated. A 1800 A-M Systems headstage (A-M Systems, Washington, USA) was used to provide a low-noise link between the recording electrode and a 1800 A-M Systems microelectrode amplifier (A-M Systems, Washington, USA). Signals were digitized at 24.4 kHz using the analog input of a RZ2 TDT multichannel recording system (Tucker Davis Technologies, Florida, USA).

A penetrating electrode array ICS-96 (Blackrock Microsystems, Utah, USA), spanning an area of 4 × 4 mm^2^, was then used to record cortical activity following retinal stimulation. The electrodes were arranged according to a square 10 × 10 grid with a lateral electrode-electrode separation of 400 μm. The four corner electrodes were disabled, leaving a total of 96 active electrodes. Tissue damage was minimized by a rapid insertion of the electrode array into the brain using a pneumatically-actuated inserter (Blackrock Microsystems, Utah, USA). The electrode array was interfaced to a PZ5 neurodigitizer (Tucker Davis Technologies, Florida, USA), which was in turn optically connected to the RZ2 TDT system. Signals were also sampled at 24.4 kHz per channel.

#### Animal Preparation and Surgery

Four sighted adult cats, two males and two females, of postnatal ages between 435 and 1600 days (median 864 days), and weighting between 3.37 and 4.26 kg (median 3.90 kg) were used in this study. Anaesthesia was induced using an anaesthetic chamber with 3–4% of isofluorane in oxygen and maintained by inhaled isofluorane (1.5 to 3.0% in air) as described previously^23^. A tracheostomy was performed and the femoral vein and artery were cannulated for drug delivery and monitoring of vitals (arterial blood pressure and heart rate) respectively. Afterwards, the anaesthetic regime was changed to a combined constant intravenous infusion of alphaxalone (2.0 to 4.0 mg/kg/h in 10 mL solution with 20 mL of 5% glucose and 20 mlL of Hartmann’s solution) and inhaled isofluorane (0–3% in equal parts of oxygen and air). The animal was placed under mechanical ventilation to ensure adequate levels of humidified oxygen and anaesthesia while monitoring end-tidal CO_2_ levels throughout. Core temperature was continuously monitored using a temperature probe and regulated using a close-loop controlled thermal blanket and an air-filled cocoon, both placed beneath the animal. Daily administration of dexamethasone (intramuscular, 1.5 mg/kg), enrofloxacin (intramuscular, 0.1 ml/kg), and atropine (subcutaneous, 0.2 mg/kg) prevented inflammation, infection and secretions in the airways respectively.

Suprachoroidal insertion of the retinal electrode array was performed though a scleral incision 7 mm posterior to the limbus. Correct positioning of the stimulating array was aided by depth markers and verified using two image registration methods: infra-red fundus imaging (in-house-built system) and optical coherence tomography (Bioptigen, Morrisville, NC, US), as shown in Fig. 7. Tropicamide and atropine eye drops facilitated pupil dilation, and corneal hydration was maintained by topical administration of hydroxypropyl methylcellulose ophthalmic gel.

After placing the animal within a stereotaxic frame, a craniotomy and durotomy were performed to expose the contralateral primary and secondary regions of visual cortex following coordinates from Tusa *et al*.^47^. The background luminance was maintained below 3.0 lux during each experiment.

#### Cortical mapping and stimulation parameters

Mapping of the visual cortex was achieved using the platinum ball electrode while biphasic (cathodic first) constant-current pulses, 500 μA in amplitude and 500 μs per phase and 10 μs inter-phase time, were delivered though the retinal electrode array. Only one electrode was activated at each time during mapping of the visual cortex, and was connected to the first channel of the neurostimulator using the switch matrix. Both the retinal and the cortical locations providing the highest evoked response, typically in the proximity to the area centralis, were identified by computing trial-average evoked potentials in a total of 50 repetitions. The penetrating electrode array was then inserted in that cortical region and stimuli were delivered to the corresponding retinal area.

Next, seven electrodes, arranged within the hexagon centred at the retinal area identified in the previous step, were simultaneously stimulated using charge-balanced biphasic, symmetric, constant-current pulses with a phase time of 400 μs (cathodic first) and an inter-phase interval of 10 μs. Each electrode was connected to an independent current source whereas all active sinks were connected to the monopolar return electrode, as shown in Fig 7. The amplitude was randomized using between 0 and 600 μA with a resolution of 50 μA. With each electrode having an area of 0.113 mm^2^, the maximum current injected provided a maximum charge density of nominally 212 μC·cm^−2^. Each stimulus was repeated 50 times leaving a 1 second gap between two consecutive stimuli. Electric current was returned through a large platinum plate electrode, 3.5 mm in diameter, placed on the surface of the sclera. At the end of each stimulus, all electrodes were briefly shorted to each other to recover any residual charges present and to ensure a common starting potential for subsequent stimuli.

#### *In vivo* data analysis

*In vivo* electrophysiological recordings were analysed off-line using custom scripts in Matlab *2013b (The MathWorks, Massachusetts,* USA). Following the algorithm described by Heffer and co-workers^48^, the stimulus artefact was removed by linear interpolation of the recorded voltage values 2 ms pre- and post-stimulus. Cortical recordings were band-pass filtered between 1 kHz and 7 kHz using a zero-phase fifth-order Butterworth filter. For each channel, the root mean square (RMS) voltage was then calculated using a period of signals taken from 100 ms preceding up to the point of stimulus delivery. Multiunit spikes were identified using a threshold level 3.8 times the RMS value of each channel within a time window between 2 ms and 15 ms after stimulus delivery. This time interval was considered sufficient to account for direct activation only of RGCs^32^. Following a conservative approach, the spontaneous spiking rate was deemed as the median spike rate of all channels when no stimulus was delivered. A responsive channel was then classified in this analysis if it reached at least 1.8 times the spontaneous spiking rate following electrical stimulation. Responding channels were further classified, based on the work done by Woolley and Casseday^49^, according to:

- Monotonic, if the Pearson’s correlation coefficient was greater than 0.75.
- Saturated, if the coefficient of determination was greater than 0.75 when performing a least-squares fit of the spike rate to equation 1,
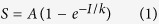
where *A* represents the gain, *k* represents the decay constant of the exponential term, and *S* and *I* are the spike rate and stimulus amplitude respectively.
- Non-monotonic, if a drop of 10% below maximum spiking rate was observed in the average of the following four stimulation levels after the maximum value.

Subsequently, the ratio of each type of response was calculated relative to the total number of responding channels per subject. The P_50_ activation threshold, established as the mid-point of a sigmoidal regression curve^15^,^23^, was calculated to the average of all responding channels and to the average of saturated channels. Results were compared using one-way ANOVA analysis and considered statistically significant at the 95% confidence level.

### *In vitro* electrophysiology

Four Wild-type C57BL/6 mice aged 4–8 weeks were anesthetized with isofluorane and euthanized by cervical dislocation. The eye was hemisected and the retina was isolated and cut into four sections. One retinal section was placed onto a membrane insert and a cut 1 mL syringe was pulled at the opposite side of the membrane insert to secure the retinal section as described by Toychiev *et al*.^50^. The retinal section along with the membrane insert was placed into an imaging chamber (Warner Instruments, Hamden, CT, USA) and was perfused with Ames’ medium equilibrated with 5% CO_2_ and 95% O_2_ to pH 7.4 and heated to 33–35 °C at 3–4 mL/min.

Electrophysiological responses were obtained by whole-cell patch clamp recording. A patch pipette was attached to a RGC, and the RGC membrane was broken through to record spiking responses using a SE as described by Tsai *et al*.^33^. Single monophasic cathodic pulses were delivered at 1 Hz with pulse duration of 400 μs through a 25-um-dimeter electrode. The stimulation amplitudes ranged from 5 to 120 μA in steps of 5 μA. Three trials were repeated for each stimulation amplitude. Once a RGC was patched, the SE was placed 20 μm above the cell epiretinally. All SE positions were recorded and controlled from a Sutter controller display panel (Sutter instrument, Novato, CA, USA).

The amplitude of the largest spike was identified and used to define a reference threshold. APs were then deemed to occur if the amplitude of a given spike was at least half of that of the largest amplitude. A 100% elicitation rate (three spike elicitations out of three trials for a given stimulation amplitude) was considered as the criterion to denote a full AP^51^. Similarly, an AP was considered to be inhibited if no spike was elicited in all trials for a given stimulation amplitude (100% inhibition rate). Multi-unit activity was obtained by combining spikes from neighbouring positions. All responses were classified as monotonic, saturated or non-monotonic as previously defined in the *in vivo* electrophysiology.

### Finite element electric field analysis

The FEM was applied to solve an electrostatic model of the eye ball and a stimulating hexagonally arranged electrode array using COMSOL Multiphysics (COMSOL AB, Sweden). The eye was assumed to be an ideal sphere, 12 mm in radius, and composed of an outer retinal layer, 400 μm thick, and an inner core defining the vitreous. A single hexagon, with identical geometry to that used in the *in vivo* experimentation, was embedded onto the suprachoroidal surface of the retina. A distal return electrode 3.5 mm in diameter was placed onto the retinal surface directly opposite to the stimulating electrodes, and set as ground. The bulk retinal tissue was assumed to be electrically passive, with its extracellular voltage distribution V_e_ (V) governed by Poisson’s equation, as in equation 2:


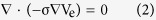


A large range for the extracellular resistivity of the retinal layer (from 500 to 7900 Ω*∙*cm) has been reported^52^. Provided that this parameter in FEM model does not influence field summation effects, its value was chosen in agreement with previous studies^53^. The resistivities of the retina and vitreous, denoted in equation 2 by 1/σ, were set to 5000 and 78.1 Ω*∙*cm respectively^53^, and assumed to be uniform and isotropic within each layer. A floating potential boundary condition was imposed to model current injection, as in equation 3,


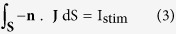


where **n** is the outward normal to the electrode boundary, dS is an infinitesimal area element of the electro boundary S, and I_stim_ is the applied stimulus current (200 μA). The geometry was meshed using tetrahedral elements, with element refinement at the electrode boundaries, and quadratic Lagrangian element shape functions were employed to solve the model. The total degrees of freedom solved for was 3,137,658.

### Computational RGC layer

A population-based RGC model was implemented in NEURON computational software^54^. The RGC cluster was reconstructed by using 39 × 39 multi-compartment RGCs over a two-dimensional plane with fixed distance between neighbouring somas, as shown in Fig. 6. The biophysical properties of each RGC were represented by Hodgkin-Huxley type equations and included sodium (I_Na_), calcium (I_Ca_), delayed rectifier potassium (I_K_), A-type (I_KA_), and calcium-activated potassium (I_CaK_), currents, hyperpolarization-activated current (I_h_) low-threshold voltage-activated calcium (I_CaT_) and leak (I_L_) currents, as in equation 4:


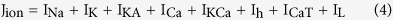


The RGC had a dendritic field diameter of 208 μm, and stratified at a depth of ~90% in the inner plexiform layer (the edge of the ganglion cell layer being 0%). Detailed morphological properties of each neuron were included in the model (see Fig. 6). In particular, a compartmentalized axon of 0.94 μm diameter and 1000 μm length was connected to the soma, and contained an axonal initial segment (AIS) of 0.94-μm diameter and 40-μm length, followed by an axon hillock of 40-μm length. All RGC model parameter settings as described by Guo *et al*.^55^. Membrane capacitance per unit area was set to 1 μF∙cm^−2^. The intracellular axial resistivity was set to 55 Ω∙cm in order to better match the *in vivo* experimental data.

A single circular electrode was stimulated relative to a distant monopolar return. The surface area, 0.791 mm^2^, was chosen equivalent to that of the hexagonal unit electrode array that was used for the *in vivo* experiment. The tissue was considered to be electrically homogeneous. The extracellular potential V at each point was approximated by the equation 5, as previously reported^39^:


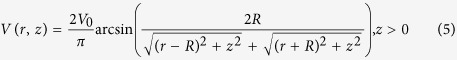


where r and z are the radial and axial distance respectively from the centre of the disk, R is the radius of the disk (502 μm), and V_0_ is the disk potential. If the extracellular space is assumed to be purely resistive, with electrical ground located infinitely far away, then V_0_ can be determined from the applied current, as given in equation 6^52^,^56^:


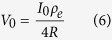


where I_0_ is the stimulation current and ρ_e_ is the extracellular resistivity (ρ_e_ = 2100 Ω.cm). The stimulation electrode was placed suprachoroidally 400 μm above the RGC array (Fig. 6).

Simulated RGC somatic responses were measured within the 15 ms time window after the stimulus offset. The RGC model did not exhibit spontaneous firing, and therefore all spikes were assumed to be stimulus induced. The stimulation charge densities in the model were extended from a maximum of 212 μC·cm^−2^ (*in vivo*) to 350 μC·cm^−2^. Finally, responses from different sub-regions within the RGC cluster were analysed and classified following the same criteria as per the *in vivo* and *in vitro* experiments.

## Additional Information

**How to cite this article**: Barriga-Rivera, A. *et al*. High-amplitude electrical stimulation can reduce elicited neuronal activity in visual prosthesis. *Sci. Rep.* **7**, 42682; doi: 10.1038/srep42682 (2017).

**Publisher's note:** Springer Nature remains neutral with regard to jurisdictional claims in published maps and institutional affiliations.

## Figures and Tables

**Table 1:** For each cat, the table shows the total number of responding channels, the number (Num) and the percentage (P) of those classified as monotonic, saturated, and non-monotonic.

CAT	Total	Monotonic		Saturated		Non-monotonic		Non-classified	
		Num	P (%)	Num	P (%)	Num	P (%)	Num	P (%)
1	45	12	26.7	10	22.2	14	31.1	9	20.0
2	92	16	17.4	16	17.4	45	48.9	15	16.3
3	43	9	20.9	13	30.2	17	39.5	4	9.3
4	51	14	27.4	6	11.2	26	51.0	5	9.8

Additionally, those which were not allocated to any group were labelled as non-classified.
